# Viral infections that alter estrogen levels during pregnancy may contribute to the etiology of cryptorchidism

**DOI:** 10.1186/s12610-021-00135-7

**Published:** 2021-07-08

**Authors:** Faruk Hadziselimovic

**Affiliations:** Cryptorchidism Research Institute, Children’s day care center Liestal, Liestal, Switzerland

**Keywords:** Cryptorchidies, estrogène, épididymes, hypogonadotrope hypogonadisme, Zika virus, Cryptorchidism, Estrogen, Epididymis, Hypogonadotropic hypogonadism, Zika virus

## Abstract

Cryptorchidism is as common as type 2 diabetes or celiac disease. Boys with congenital cryptorchidism are at increased risk of infertility and testicular cancer. Zika syndrome, which affects pregnant women, is associated with a high incidence of undescended testes in the infant, accompanied by epididymal anomalies. Zika and influenza virus infections during pregnancy trigger a strong anti-inflammatory immune response and elevated estradiol levels. Elevated estradiol and α-fetoprotein in syncytiotrophoblasts from women who have given birth to cryptorchid boys are indicative of increased estradiol levels in the fetus. Here, I present a hypothesis that hypogonadotropic hypogonadism, cryptorchidism, and retarded epididymal development may be due to elevated fetal estradiol levels caused by viral infection during pregnancy.

## Résumé

La cryptorchidie est. aussi fréquente que le diabète de type 2 ou la maladie cœliaque. Les garçons atteints de cryptorchidie congénitale ont un risque élevé d’infertilité et de cancer des testicules. Le syndrome Zika, qui affecte les femmes enceintes, est. associé à une incidence élevée de testicules non descendus, accompagnée d’anomalies épididymaires, chez le nourrisson. Les infections virales Zika et Influenza déclenchent une forte réponse immunitaire anti-inflammatoire et des taux d’estradiol élevés pendant la grossesse. Des taux élevés d’estradiol et AFP (α-fetoprotein) dans les syncytiotrophoblastes de femmes qui ont donné naissance à des garçons cryptorchidies indiquent une augmentation des taux d’estradiol chez le fœtus. Ici, je propose l’hypothèse que les effets secondaires potentiels d’un taux élevé d’estradiol fœtal sont l’hypogonadisme hypogonadotrope, la cryptorchidie et le retard du développement épididymaire.

## Introduction

Cryptorchidism is the most common congenital disorder in newborn boys, affecting 1–3% of full-term newborns [1]. This birth disorder is a major risk factor for male infertility and testicular malignancy during adulthood.

Zika syndrome is a unique pattern of anomalies and disabilities found in children infected with the Zika virus in utero, and 36.4% (8/22) of infected boys present with cryptorchidism. In this context, cryptorchidism has been hypothesized to be an additional malformation associated with congenital Zika syndrome. Intriguingly, boys with cryptorchidism have a high prevalence of testis-epididymis dissociation (55.6%), which suggests a link between the phenomena of undescended testis and abnormal epididymal development [2, 3]. Mice treated with estradiol are protected against intravaginal Zika virus infection independent of interferon (IFN)-α/β or IFN-λ signaling. Exogenous IFN-λ treatment has been shown to confer an antiviral effect in mice given both estradiol and progesterone, but not in those that receive progesterone alone [4]. In another study, 12% of 316 boys with congenital rubella presented with cryptorchidism, and the epididymal system was absent or apparently obstructed in 60% of the cases [5]. Furthermore, pregnant women infected with influenza virus during the second and third trimesters have an increased risk of severe cardiopulmonary complications, premature delivery, and death [6].

Pregnancy-related levels of 17-β-estradiol can induce key anti-inflammatory cell phenotypes in the immune response to a virus, independent of other hormones or pregnancy-related stressors [6]. Thus, elevated estrogen levels may result in an attenuated anti-viral immune response, and pregnancy-associated morbidities.

### Cryptorchidism may be a consequence of viral infection during pregnancy

**Fig. 1:**
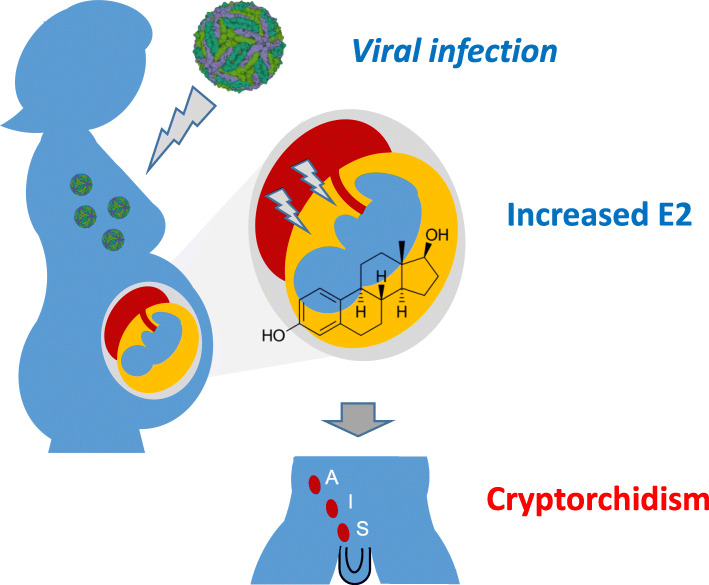
Schematic of the model postulating a viral etiology for post-natal cryptorchidism. The infectious agent shown is a model of Zika virus from the Protein Databank (www.rcsb.org/structure/5IRE). E2, 17-β-estradiol; S, pre-scrotal cryptorchidism; I, inguinal cryptorchidism; A, abdominal cryptorchidism

In the placenta of women who give birth to normal males, estradiol weakly accumulates, predominantly in the basal region of the syncytiotrophoblast in the terminal placental villi. In contrast, all placentas from mothers of males with cryptorchidism exhibit high estradiol levels in the basal portion of the syncytiotrophoblast [7]. Importantly, we employ stringent criteria to diagnose cryptorchidism. Undescended testes diagnosed at birth are regularly checked until orchidopexy is carried out, and testicular biopsies are collected. Histological examination of semithin sections prove that cryptorchid, not retractile testes, were treated and analyzed. Finally, elevated levels of serum α-fetoprotein (AFP) are found in the placenta [8] or serum [9] of pregnant mothers with cryptorchid boys, supporting the hypothesis of an estrogenic origin. AFP is thought to mediate the response of the developing fetus to estrogen [9]. Interestingly, mothers who carry fetuses with cryptorchidism have significantly elevated percentages of free and albumin-bound estradiol in the serum during the first trimester [10]. Based on these observations, I hypothesize that viral infection may induce hypogonadotropic hypogonadism and cryptorchidism by altering 17-β-estradiol levels (Fig. 1).

### Evidence in support of the estrogen hypothesis

Currently, the specific cause of isolated cryptorchidism is unknown in most cases, but indirect evidence suggests that the disease is likely influenced by multiple processes. It has been reported that up to 70% of boys with cryptorchidism suffer from hypogonadotropic hypogonadism [11], whereas the remaining cases may represent boys with ascended testes.

Reduced fibroblast growth factor expression may explain the reduction in prokineticin 2 (*PROK2*) gene expression in samples from boys with cryptorchidism. This reduction induces central hypogonadotropic hypogonadism, which is characterized by low basal and stimulated luteinizing hormone (LH) [12, 13] and impairs epididymal mesoderm development, which results in abnormal descent of the epididymal-testicular union [14, 15]. Except for a blunted testosterone response to human chorionic gonadotropin (hCG), there is no evidence of altered steroidogenesis in cryptorchid testes prior to puberty [16]. When boys with cryptorchidism are treated with hCG, their response to a stimulation test is no longer different from the one observed in a control population [17]. Thus, the cause of the low testosterone response appears to be at the pituitary or hypothalamic level. Numerous tests with LH-releasing hormone have demonstrated that the LH response is abnormally low in boys with cryptorchidism [18].

When pregnant female mice are treated with estradiol, the male progeny exhibit hypogonadotropic hypogonadism with low pituitary LH and low testicular testosterone, resulting in abnormal epididymal development and cryptorchidism [19–21]. Both gonadotropin releasing hormone agonists and hCG treatment for estradiol-induced cryptorchidism prevent abnormal development of the epididymis and induce complete descent in 60% of undescended gonads [19–21].

Pregnancy-related estradiol levels have shown divergent effects on the host immune response against H5N1 influenza virus infection. The anti-inflammatory properties of estradiol attenuate infection-associated lung inflammation in mice and the production of virus-specific antibodies after an infection [22]. In general, viral infections in pregnant women trigger an immune response that leads to an increased concentration of 17-β-estradiol in the syncytiotrophoblast, which may affect the sexual development of male fetuses.

Interestingly, the incidence of cryptorchidism is significantly higher during spring (February-April; 3.0%) than summer (May-July; 1.7%) [23]. This seasonal difference has been observed among both preterm and term boys. Thus, a seasonal fluctuation in cryptorchidism incidence is likely due to environmental factors [24]. A proposed cause of this variation and its relationship to the etiology of cryptorchidism is low exposure to light during the dark winter months [24, 25].

Kristensen et al. argued that intrauterine exposure to mild analgesics is a risk factor for the development of male reproductive disorders [26]. However, a systematic review and meta-analysis suggested that analgesia use during pregnancy is not strongly associated with the development of cryptorchidism [27]. Importantly, median paracetamol clearance is significantly higher at delivery than in postpartum or non-pregnant women, but an association between paracetamol clearance and estradiol has been observed (*R* = 0.494, *p* < 0.0001) [28]. Thus, using paracetamol as a treatment for viral infections, which predominately occur during the winter months, induces a dose-dependent increase in estradiol that could possibly interfere with testicular descent. The viral model proposed here offers an alternative explanation of a higher rate of viral infections during the colder period of the year.

## Conclusions

The model presented here postulates virus-induced endocrinological effects on male sexual development and could explain a potentially critical component of cryptorchidism. This proposed model is consistent with some of the relevant physiological and seasonal data and applies to all viral infections that affect estrogen levels in placental cells. Furthermore, the hypothesis emphasizes the importance of determining endocrinological parameters during the clinical diagnosis of viral syndromes to avoid long-term effects on fertility and testicular malignancies. Further work is warranted to test this model of the contribution of viral infections to the etiology of cryptorchidism.

## Data Availability

Not applicable.

## Abbreviations

AFP: Alpha-fetoprotein
LH: Luteinizing hormone
HCG: Human chorionic gonadotropin
PROK2: Prokineticin 2
